# Bibliographical Notices

**Published:** 1841-03-06

**Authors:** 


MEDICAL EXAMINER.

DEVOTED TO MEDICINE, SURGERY, AND THE COLLATERAL SCIENCES.

No. 10.] PHILADELPHIA, SATURDAY, MARCH 6, 1841. [Vol. IV.

### BIBLIOGRAPHICAL NOTICES.

*Recherches Anatomiques, Pathologiques et Thérapeutiques, sur la maladie connue sous les noms de Fièvre Typhoïde, &c.* Par P. C. A. Louis. Deuxième Edition, considérablement augmentée. A Paris: 1841.

*Remarks, Anatomical, Pathological, and Therapeutical, on the disease known under the name of Typhoid Fever, &c., compared with the most frequent acute diseases.* By P. C. A. Louis, Physician to the Hôtel Dieu, Perpetual President of the Society of Observation, &c. Second Edition, considerably increased. 2 vols. pp. 542—523. Paris, 1841.

(Continued from page 140.)

Convalescence took place, on an average, ten days after the admission of the patients into La Pitié, and nineteen days after the appearance of the first symptoms.

The treatment of these patients offered nothing remarkable. The Seidlitz water was administered to them in the manner before indicated; and, on an average, during five days. It was replaced, once only, in one patient, by forty-five "grammes" of castor oil. The habitual drink was the solution of tartaric acid syrup, or the citric lemonade in a larger or smaller quantity.

In one case where the cephalalgia was violent and obstinate, recourse was had to an application of leeches to the mastoid apophyses, and as is usually the case, without evident success.

The cases just detailed, adds M. Barth in concluding, were equally well distributed among the different months of the year, to remove all idea of epidemic constitution; so that, from this account, these facts are entirely comparable with those which we have just treated of, and particularly with those which relate to cases treated by myself at La Pitié, by means of moderate bleedings, Seidlitz water and injections; and which, it will be recollected, amount to a hundred, of whom twelve died, or a little less than an eighth.

This last result is less favourable than that which followed the action of purgatives, in those patients whose histories I have just analyzed; without, however, our being able to conclude, rigorously, as to the superiority of evacuants, on account of the small number of cases treated by them, inasmuch as one subject more among these patients could have produced one more death, and consequently have changed in a remarkable manner the mortality, which would then have been an eighth.

Nevertheless, these two series of facts appear to me to be valuable, inasmuch as the proportion of light and grave cases is pretty equal in both; and in both the patients were treated in the same hospital, in the same wards, and by the same physician; that, indifferent to the results which should follow the administration of this or that therapeutic means, I was not drawn into error by an admitted preference, to the employment of such or such of them.*

If we can conclude rigorously from the preceding facts, even from those collected and exposed by M. De Larroque, on the superiority of evacuants in the treatment of typhoid fever, we must at least acknowledge, for nothing is plainer, that these agents have not the harmful effects attributed to them, for a long time, on the course and termination of this disease; that we can administer them without fear; that it is even quite probable, from the facts alone collected by Dr. Barth, at La Pitié, in my division, that these agents are superior to the other therapeutic means. And the conclusion would be entirely certain, if these facts, and those communicated by M. De Larroque to the Royal Academy of Medicine, were more numerous, and if this physician had entered into a greater number of details concerning them. It is evidently also to the study of the action of evacuants on the course and termination of typhoid fever, that practitioners should now especially attach themselves.

I would add that with the exception of M. De Larroque, the physicians who have employed evacuants in the treatment of typhoid fever, have not used at all, or very rarely the emetic tartar at the commencement; that this omission may have modified the cipher of mortality; that if men, learned and profoundly versed in their knowledge of typhoid fever,

* It is also probably to the small number of patients treated by purgatives, that we owe the rather remarkable differences found in the duration of the disease in the grave cases, in the patients treated by purgatives, and in those treated at La Charite by bleeding, (two small bleedings in the first ten days of the affection.) Under the influence of this last treatment, in fact, the average duration of the disease was thirty days, in the grave cases, and twenty-five in the light ones; while here, under the influence of purgatives, this duration was, as has been said, in the grave cases, in those of middling gravity, and in the light cases, thirty-four, twenty and nineteen days.

have proscribed the use of purgatives at that stage of the disease in which we can admit the ulcerations of the small intestines, on account of the possible influence of its contractions on the formation of perforations, their principles should bow before experience, which shows that this accident is not more to be feared, that it is perhaps less to be dreaded in the evacuant than in the antiphlogistic treatment. In fact, in the thirty-two cases of typhoid fever, treated by me at La Pitié, by means of purgatives, I did not see one example of this lesion. In fifty other patients whom I treated in the same manner, and at all periods of the disease, at the Hôtel Dieu, last year, I met with only one case of perforation; and MM. Andral and Piédagnel are silent on this point.

It will, perhaps, be said, that an epidemic of typhoid fever supervening, could show the vanity of all that I have said favourable to the employment of purgatives. I would answer, that we do not here consider epidemics, but only the typhoid affection observed in a sporadic state; that we must admit that in analogous conditions, the treatment which succeeded at one period, in a pretty considerable mass of persons, would succeed again; without which there would be no possible therapeutics, and experience would be a vain word.

Now, in admitting as demonstrated the superiority of evacuants, in the treatment of typhoid fever, considered in a general manner, should we employ them in all cases indistinctly, whether they be grave or slight; whatever be the predominance of the symptoms, in the ataxic and adynamic forms? The answer to these questions appears easy to me. On one part, the degree of a disease does not change the nature of its treatment; it is only necessary to follow it with more or less energy. Again, if the mortality of the disease is less under the influence of evacuants than under that of the other methods of treatment, it can only be by modifying more advantageously the grave cases than these last means; perhaps, also, certain slight cases, which become pretty often fatal, as has been seen, by perforation of the small intestines, when the patients are treated by the means usually employed. So that in admitting the superiority of evacuants in the treatment of the typhoid affection, considered in the generality of cases, it is plain that these agents should be employed in severe as well as slight cases,—only with more or less energy.

As to the forms called ataxic and adynamic, the most severe of all, those in which the mortality is greater, which furnish the greatest number of these unfortunate cases, it is still very evident that the evacuant treatment is principally applicable to them, if, as we admit provisionally, this treatment is generally more successful than the others; and it is not presumed that the ataxic form, which is the most fearful, calls for any great modifications in this respect, provided that it is not ordinarily accompanied by appreciable lesions of the encephalon, and that diseases which, like pneumonia, for example, are frequently accompanied by cerebral symptoms, get well by the same treatment, whether these symptoms do or do not exist. We will see, however, in the following chapter, what modifications it would perhaps be well to join to the evacuant treatment, as it has been described, when the ataxic symptoms develope themselves.

These are strong arguments in favour of purgatives; and Dr. Louis, conscientious as he is, evidently leans strongly to that method of practice, although it was one which he has only lately adopted. But, it must be remarked, that these purgatives are mild, and very different from the drastics, which are almost always pernicious. The treatment is not, however, perfectly settled, for typhoid fever is essentially variable as to its mortality, and if the prevailing tendency of the disease is to the milder types, it will necessarily give a trifling average. As to purgatives in mild cases, our views are, that they should always be given at first for two or three days, and that they should be of the mildest kinds, as a few grains of blue mass, followed by oil or Seidlitz powder; if the disease then go on regularly, and offer no peculiar symptoms, we are disposed to let it take its own course, and we restrict ourselves to a careful regulation of the diet, which should be as mild as possible, and to the mild diuretics, such as neutral mixture, &c. Even the latter remedies, in some regular cases, appear perfectly unnecessary. If constipation should occur, a repetition of the laxative is always of benefit. The most troublesome symptoms during the course of these mild cases are the occasional increase of cerebral symptoms, as dizziness and cephalalgia; a cupping to the head, or even a mustard pediluvium, will often dissipate, or greatly relieve these symptoms. A blister to the nucha produces a very similar effect; and we have never seen any disadvantage result from this mode of using it, in which particular we differ from Dr. Louis, who forbids blisters in all cases. It is true, that the benefits of them, when applied to the legs, are by no means evident, and they will occasionally pass into gangrene. But we have never seen any marked injury result from these applications to the nucha; and if properly used—that is, at the stage of the disorder when the acute febrile symptoms have declined,—they rarely fail to relieve the slight stupor and delirium.

In the treatment of the severe cases of the disorder, we do not consider the question as to the propriety of purgatives, as a settled one. We cannot agree with our author that the treatment of the disease should be necessarily the same in bad and in slight cases, and that it should be merely rendered more energetic in the former cases; this would completely unsettle our notions of therapeutics, and it is especially inapplicable in diseases such as typhoid fever, which have a natural duration. Now in those whose duration is not limited, the rule admits of numerous exceptions,—for instance, dysentery, in which the remedies appropriated to one stage or variety of the disorder are totally unlike those adapted to another. In typhoid fever the difficulty of adapting remedies to particular cases, is extremely great, and we are often in doubt as to the best mode of managing those of great severity. In order to understand this subject, we should examine into what constitutes the severe forms of the disease. The fever is severe chiefly because the cerebral symptoms, such as delirium, stupor, subsultus, &c., are highly developed, this state of things is generally accompanied with a tendency to sloughing, and the whole train of symptoms connected with decided alteration of the blood. The disease may also become severe from the increase in the symptoms of any particular organ, such as the lungs or the bowels, the patient may then labour under pneumonia or severe diarrhœa. In the latter case the indications for treatment are well established; but in the former there is much difficulty, especially if the disease be complicated with either excessive diarrhœa or the affections of the lungs, and we do not think it possible to lay down any uniform method of treatment.

We cannot positively condemn evacuants, but we believe they should be restricted to the mildest laxatives, such as oil, and there is often no apparent necessity for their administration. Our own practice is to treat such cases after they are fairly formed, by cups and leeches to the head, and diaphoretics, with revulsives to the lower extremities, of a kind that do not vesicate—that is, weak mustard poultices, and hot applications; wine, or stimulants of any kind, we do not prescribe, unless there should be a decided tendency to sinking. This treatment, it is evident, cannot be called active, nor do we conceive that this is necessary or beneficial in the treatment of the disorder. We refrain, however, from expressing a positive opinion as to the propriety of using purgatives in these cases, leaving that as a question to be decided only by future observers.

In both severe and mild cases of typhoid fever, alteratives are beneficial towards the decline of the disease,—that is, about the end of the second week if the disease be severe, or a little earlier if it be mild. The best indications for their use are the same as are admitted in most acute diseases,—that is, a coated tongue, especially if a little disposition to clean is observed. The use of this class of remedies in acute diseases is scarcely known in France, and we believe that they lose a most efficient therapeutic means. The alteratives which we prefer, are small doses of calomel or blue mass, with ipecacuanha, as one grain of the latter, and a quarter of a grain of the former, with half a grain or a grain of ipecac.; a little opium may be added if there is a tendency to much diarrhœa. A moderate discharge from the bowels,—that is, once or twice daily, is of service in all stages of the disease, and should on no account be interrupted.

The oil of turpentine is proposed as a remedy by Dr. Wood, of this city, especially when the tongue has begun to clear, but remains smooth and dry; in our practice, we have found the remedy uncertain—at times of great benefit, and at others of little advantage, or even increasing the dryness of the tongue.

Towards the close of the severe cases, tonics, such as the sulphate of quinine, and wine whey, are often necessary, but in the early part of the disease they are almost always of mischief. Opium is also often applicable in the typhoid, just as in typhus fever, when the patient remains very restless, and the more acute symptoms have passed off. It should not, in general, be given in more than half the usual dose.

After having examined the effect of different therapeutic agents in particular, Dr. Louis passes to some remarks on "the treatment in general."

It is very clear, says our author, that the treatment by purgatives is not proven incontestably, and it cannot be recommended to the exclusion of blood-letting. If the latter mode of treatment should be preferred, it may be employed as follows:

The bleeding should be moderate, (ten or twelve ounces,) and once or twice repeated, according to the strength of the patients; a frequent repetition is worse than useless. After the first fifteen days in mild cases, or before that time in slight ones, the bleeding retards rather than hastens convalescence. The influence of blood-letting should be promoted by diluents, a moderate temperature, and mucilaginous enemata. The drinks should be slightly acid: the best acid is the carbonic. A weak solution of chloride of soda may often be used in place of the acidulous drinks. If the diarrhœa exhaust the patient, it should be checked by injections of laudanum. If the purgative treatment is preferred, the physician should give at first a little tartar emetic, largely diluted, to produce emesis, and afterwards give every day, or every second day, some Seidlitz water, or Pullua water, (Epsom salts would do as well,) or oil or calomel enough to produce four or five stools daily. If hypercatharsis ensue, injections of laudanum may be given.

The hygienic precautions of ventilation and avoiding pressure on certain portions of the body, should also be insisted upon. Towards the conclusion of the disease some tonics may be given, either mild or more powerful, according to the condition of the patient.

In severe cases, says Dr. Louis, blisters are merely a new disease added to the former, nor does it seem that points of irritation established by other means, such as sinapisms, can be useful. From this opinion we have already expressed our formal dissent.

As the delirium is not explained by the condition of the brain, and as the application of ice did not seem of any decided benefit, our author forbids it: we do not quite agree with him. Ice is sometimes of benefit; cold cloths, dipped in cool water, almost always are, if there be any appearance of congestion or heat of the head. A moderate bleeding is advised in these cases of delirium, or a continuance of purgatives, if they were previously employed.

Tympanites cannot be controlled with any great certainty of success. We would recommend, with the author, mucilaginous injections, adding, at times, a portion of assafœtida. The spasms of muscles may be combated with opium, especially in the form of injection. We prefer camphor for the same purpose. In perforation of the intestine, very large doses of opium offer the only chance of recovery.

The retention of urine requires the usual surgical attentions. Inflammation of the throat may be relieved by mucilaginous gargles,—the purgative treatment must be suspended if symptoms of irritation of the stomach should supervene. The cough rarely requires any special remedies, except it be a sign of pneumonia, in which case Dr. Louis recommends bleeding at the early stages, and the contra-stimulant treatment in the latter, if the patient is not extremely exhausted. We should hesitate very much as to this matter. The cold infusion of bark is recommended in convalescence to prevent profuse cold sweats, and opiates for removing the sleeplessness occurring frequently at the same period.

The typhoid fever of young children does not require a very different treatment from that already directed; at least the modifications are slight, and depend mainly upon the age of the patients.

We have laid the greatest stress upon the additions made by Dr. Louis to the treatment of typhoid fever, not that the other matter is unimportant; but it is rather carrying out what he had already published, than adding what is positively new. It is evident to our readers that the subject which is most wanting in demonstrative proof is the treatment of the disorder; the same is, to some extent, true in most febrile affections having a self-limited duration. But we may consider the treatment as established in most respects; the disease is rather of an inflammatory character, which is at times badly marked; hence the treatment at the commencement should not be stimulating; it may become so late in the disorder, when the blood is decidedly altered. Remedies which favour the natural secretion, whether mild alteratives, diaphoretics, or laxatives, are useful, with nervous counter-irritants, if the brain be much affected.

The disease itself has now become a familiar one to American practitioners, for which they are in great part indebted to the reviews and to the translation by Dr. Bowditch of the former edition of Dr. Louis's work. This must remain a monument to the author; and we may hope that the zeal and affection of Dr. Bowditch towards his teacher will induce him to complete the translation by adding the important new matter as a supplement to the former edition. It will then be a finished work.

In noticing so extended a work our task was necessarily limited by the space allotted to us. We have not selected the main body of the work, which is, beyond controversy, the most finished description of disease extant, but we have scanned most narrowly those portions which were less satisfactorily proven, and contained some assertions which we could not entirely admit; these, however, are but few in number, and are limited to those points which are of necessity thus far incompletely proven.

---

*Pathological and Surgical Observations on the Diseases of the Joints.* By Sir Benjamin C. Brodie, Bart. F. R. S., Sergeant-Surgeon to the King, and Surgeon to St. George's Hospital. Fourth edition. London.

Our notice of the late edition of the valuable work of Sir B. Brodie must be confined to the additional matter which it contains, and to the difference of opinion existing between himself and other writers of distinction, in regard to the proper classification of the diseases of which he treats. Since the publication of this work, M. Velpeau has made a special study of the same class of affections, and as the results of his observations are not yet, we believe, before the public, we shall give his views as briefly as possible, but still more in detail than would otherwise be required of us.

To Sir B. Brodie belongs the great merit of first presenting a methodical arrangement of the different diseases of the joints. These diseases had previously been confusedly grouped together under the vague term of *white swelling;* a term which in itself has no signification. Wiseman intended it to indicate any chronic swelling, without redness or alteration of colour in the skin, and not necessarily accompanied by pain. These swellings, nevertheless, frequently offered a species of fluctuation differing entirely from that which would be produced by the presence of a liquid in the cavity of the joint; they communicated to the touch a semi-elastic, doughy sensation, which gave origin to the term *fungus articuli,* as a synonyme of *white swelling.*

Both terms are faulty; the first from its insignificancy, the second because it attaches undue importance to a single symptom, which, during the early stage of articular disease, is found only in one variety, and at a later period is common to all; in fact, no expression can be found sufficiently general to include so great a variety of dissimilar affections, unless we adopt the comprehensive name suggested by Velpeau —"arthropathy," or simply disease of the joint.

The classification of these affections given by Sir B. Brodie in the first edition of his work, he has seen no reason to change; subsequent experience has in his opinion only served to confirm its correctness; he therefore still regards the different varieties of white swelling as dependent upon either inflammation of the synovial membrane—a morbid change in the structure of this membrane—ulceration of the articular cartilages—or disease of the cancellous structure of the bones. We will first contrast this arrangement with the more philosophical classification of Velpeau, and then return to our author, to analyse each of his divisions in turn. Velpeau, leaving out of consideration acute arthritis, rheumatism, &c., which would properly be included in his general term *arthropathy,* confines himself to those *chronic* forms of articular disease generally recognised as white swellings—his division of these is founded upon a difference in the anatomical seat of the lesion—and upon a difference in the general cause producing this lesion.

The tissues entering into the composition of a joint are either hard or soft, and as disease may originate in either of these, we have a natural division of white swellings into two great classes. The first embraces all those which commence in the soft parts: the second those which commence in the bones or hard parts. These two classes may be distinguished from each other, from the very commencement, by the difference of their symptoms. Every articular disease which commences in the soft parts, exhibits itself primarily by swelling, while those which commence in the hard parts first betray themselves by pain; in the sequel, both pain and swelling may co-exist in either variety, but the precedence of one or the other is sufficient to determine to which class the affection belongs.

But the soft parts themselves admit of a division into a variety of tissues; these differ in their nature, and consequently impress upon their diseases a difference in symptoms, by which we are enabled to distinguish, to a great extent, the varieties of this class. M. Velpeau divides the soft parts into inter-articular and extra-articular, and taking the knee joint for type, traces out, as far as possible, the first symptom in each of these divisions. The results of his observations are briefly these: A disease originating in the inter-articular soft parts is to be distinguished, 1st, from that commencing in the hard parts by the absence of pain while the joint is at rest;—2d, from that commencing in the extra-articular soft parts by the existence of pain in motion of the articular surfaces upon each other. In the extra-articular soft parts, little if any pain exists in those motions, which are confined to the articular surfaces, or which interest but slightly the external envelopes.

In the hard parts the disease may originate at either surface of the cartilage, or in the substance of the bone; the character of the pain differs in these several varieties, but not sufficiently to render the differential diagnosis at all positive; the very existence of the pain serves, however, to distinguish them as a class.

By this classification of Velpeau, every variety of articular disease is studied in reference to its anatomical characters. We have only to connect with it a classification founded upon the nature of the causes producing these affections, and the study will be complete. Gerdy adopts the latter as the sole basis for a division of white swellings, which he refers to four distinct causes.

*"1st, Scrofula. 2d, Rheumatism. 3d, Wounds. 4th, Eruptive fevers. That produced by scrofula, attacks first the bones; that produced by rheumatism and injuries, attacks first the surrounding soft parts; and this is occasionally true of that form subsequent to eruptive fevers, which is however of rare occurrence. This disease is common in youth, and rare after the age of forty years; in the rheumatic variety, and in that following wounds, the disease is acute; in the scrofulous variety the contrary is the case."

* Vide N. Y. Journ. Med. and Surg., January 1841—p. 219.

The general causes productive of, or predisposing to white swelling, are not, however, so limited as would appear from the paper of Prof. Gerdy. Thus gout is occasionally, although rarely, the point of departure of this disease. A woman was admitted into the hospital of "la Charité," in 1836, for a white swelling of the knee; the tumour was as large as an adult head, and was due in part to an effusion of liquid; in part to a thickening and degeneration of the tissues. She was gouty, and concretions could be felt in the knee, as well as in the ankle joint of the same limb. Brodie observes, in reference to this cause,

"There is a remarkable, yet not uncommon form of the disease, which may be considered as bearing a relation to both gout and rheumatism, but differing from them, nevertheless, in some essential circumstances. The synovial membrane becomes thickened, so as to occasion considerable enlargement of the joints, and stiffness, there being at the same time but little disposition to the effusion of fluid. In the first instance, the disease is often confined to the fingers; afterwards it extends to the knees and wrists; perhaps to nearly all the joints of the body. Throughout its whole course, the patient complains of but little pain; but he suffers, nevertheless, great inconvenience, in consequence of the gradually increasing rigidity of the joints, and the number which are affected in succession. The progress of the disease is usually very slow, and many years may elapse before it reaches what may be regarded as its most advanced stage. Sometimes, after having reached a certain point, it remains stationary, or even some degree of amendment may take place: I do not, however, remember any case in which it could be said that an actual cure had been effected. The individuals who suffer in the way which has been described, are, for the most part, those belonging to the higher classes of society, taking but little exercise, and leading luxurious lives; but there are exceptions to this rule; and the disease occasionally occurs in hospital practice—in men, and even in females of active and temperate habits."

Cancer, scurvy, and all affections capable of rendering the bones more fragile, may also be considered as occasional predisposing causes, by rendering the articular surfaces of the bones more liable to permanent injury from the effects of external violence.

Effusion into the cavity of a joint occurring after parturition, during the existence of a blenorrhagia, or from other causes, may leave a point of irritation capable of degenerating into a true white swelling. The mode of production in these cases is easily comprehended. But the observations furnished by Sir B. Brodie of the co-existence of white swelling with a gonorrheal inflammation of the urethra and conjunctiva, and, their simultaneous disappearance and reproduction, are not so readily explained—the cases are too numerous to permit us to regard them as mere coincidences, and are too well known to require further allusion to them.

The classification of Sir B. Brodie is certainly imperfect, and in some points incorrect, if we may be governed by weight of evidence; this is particularly the case in reference to his statement of the frequency of ulceration of the articular cartilages, an alteration, the possibility of which is denied by the majority of distinguished anatomists of the present day. We shall recur to this subject more at length in its proper place.

To the first division of our author, which embraces those swellings dependent upon inflammation of the synovial membrane, we find appended in the edition before us the description of a peculiar form of articular disease which is too important to pass over unnoticed.

"There is a peculiar morbid state of the system which in some instances follow severe accidents or operations, and which is well known to surgeons who are engaged in the practice of the London Hospitals, in which the patients are liable to deposits of pus in various parts of the body at a distance from the seat of the original injury. These deposits not unfrequently take place in the cavities of the joints, as a consequence of inflammation of the synovial membrane, and independently of ulceration. Several examples of the kind have fallen under my own observation, but it will be sufficient for me to refer to those who have been recorded by the late Mr. Rose, and by Mr. Arnott in the 15th vol. of the Med. Chir. Transactions.

In one of the cases related by Mr. Arnott, it is stated that the cavity of the knee joint was filled with 'tolerably thick pus, of an uniformly reddish colour, as if from an admixture of blood.'"

